# MAPPING OF ULTRASONOGRAPHY METHODS AND SHOULDER SOFT-TISSUE INJURY LOCATIONS IN PATIENTS WITH STROKE: A SCOPING REVIEW

**DOI:** 10.2340/jrm.v57.43179

**Published:** 2025-07-20

**Authors:** Masayuki DOGAN, Daisuke ITO, Shota WATANABE, Tetsuya TSUJI, Michiyuki KAWAKAMI

**Affiliations:** 1 Department of Rehabilitation Medicine, Keio University School of Medicine, Tokyo, Japan; 2 Department of Rehabilitation Medicine, Tokyo Bay Rehabilitation Hospital, Chiba, Japan

**Keywords:** shoulder soft-tissue injury, stroke, ultrasonography

## Abstract

**Objective:**

To map studies that use ultrasonography to assess shoulder soft-tissue injuries in stroke survivors and identify the methods and soft-tissue injury locations.

**Design:**

Scoping review.

**Methods:**

A literature search was performed through PubMed and ICHUSI from 1966 to May 2023 using the terms “stroke”, “shoulder soft-tissue injury”, and “ultrasonography”. Original articles that used ultrasonography to evaluate shoulder soft-tissue injuries in patients with stroke were selected. Extracted data included study design, phase, sample size, ultrasonographic methods (probe, evaluation position, frequency, and assessment site), and soft-tissue injury location.

**Results:**

Among 249 articles identified, 10 met the inclusion and exclusion criteria. In ultrasonographic methods, over half the studies used linear transducer probes, evaluated participants in a sitting position, and applied frequencies of 5–7 MHz. Common assessment sites were the supraspinatus tendon, long head of the biceps tendon, subscapularis tendon, infraspinatus tendon, and subacromial-subdeltoid bursa. The most common locations of shoulder soft-tissue injuries were the long head of the biceps tendon (effusion/tendinitis) and the supraspinatus tendon (tear/tendinitis).

**Conclusion:**

This study identified ultrasonographic methods and hemiplegic shoulder soft-tissue injury locations. These findings may help facilitate evaluations and enable proper assessment of shoulder soft-tissue injuries in patients with stroke using ultrasonography in clinical practice.

## LAY ABSTRACT

Patients with stroke often have dysfunctional shoulders, and soft-tissue injuries are one of the contributing factors. Anatomical assessment is essential for the hemiplegic shoulder in patients with stroke. Ultrasonography has the potential to be a useful and rapid method for evaluating shoulder soft tissues. However, this method has not been established and is infrequently used in stroke survivors. The present review summarizes 10 known studies in which ultrasonography was used to evaluate soft-tissue injuries in the hemiplegic shoulder. This study helps guide the evaluation of hemiplegic shoulder soft-tissue injuries in clinical practice.

Stroke is a leading cause of upper-limb dysfunction, with more than two-thirds of stroke survivors affected (1). Between 50% and 80% of stroke survivors experience upper-limb motor impairment in the acute phase, and approximately 50% of these individuals continue to have impairment 6 months post-stroke (2). Functional upper-limb rehabilitation requires control of proximal segments to position and orient the hand to the environment (3). However, the hemiplegic shoulder suffers from neurological symptoms, such as motor paresis and spasticity (4). Moreover, the shoulder is anatomically complex, and neurological symptoms can develop into anatomical disorders (5). Therefore, anatomical assessment is essential for the hemiplegic shoulder in patients with stroke.

One of the anatomical disorders affecting the hemiplegic shoulder is a soft-tissue injury. Shoulder soft-tissue injuries cause shoulder pain owing to the stretching of local neurovascular and musculoskeletal tissues (6). However, shoulder soft-tissue injuries may present with non-specific or latent symptoms, making them difficult for patients and medical staff to recognize. In orthopaedic diseases, shoulder soft-tissue injuries are significant predictors of shoulder pain (7) and are consistently evaluated in clinical settings. The simplest evaluation methods for shoulder soft-tissue injuries are physical tests, including painful arc, drop arm, supraspinatus strength, and Neer and Hawkins–Kennedy impingement signs, which are useful for rotator cuff lesions in the general population (7). However, patients with stroke who have flaccid or spastic paralysis do not have sufficient movement to participate in the physical tests (8). Furthermore, patients with stroke who have sensory impairments may be unable to perceive pain accurately during examinations (8). Therefore, the examination of soft-tissue injuries in hemiplegic shoulders after stroke remains limited.

Although static and objective evaluation methods such as magnetic resonance imaging (MRI) and arthrography are feasible for assessing soft tissue in hemiplegic shoulders after stroke, they are time-consuming and costly. Conversely, ultrasonography is a useful method for evaluating shoulder soft-tissue injuries in patients with various disorders (9). Ultrasonography can non-invasively and objectively evaluate hemiplegic shoulder soft-tissue injuries without motion and is easy to use in clinical settings (8). However, there is no consensus on the use of ultrasonography and the assessment sites for evaluating hemiplegic shoulder soft-tissue injuries in patients with stroke. The central purpose of a scoping review is to rapidly identify and map concepts that are foundational to a field of research (10) and allow the identification of gaps and areas for further research (11). In particular, scoping reviews can function as stand-alone research efforts when a particular area of research has not previously been comprehensively reviewed. A lack of comprehensive reviews exists on the use of ultrasonography and assessment sites for evaluating shoulder soft-tissue injuries in patients with stroke in clinical practice. This scoping review aimed to identify existing ultrasonographic methods and the locations of soft-tissue injuries assessed using ultrasonography in patients with stroke.

## METHODS

A scoping review was conducted in accordance with the Preferred Reporting Items for Systematic Reviews and Meta-Analyses extension for Scoping Reviews (PRISMA-ScR) guidelines (10). This study adhered to the following steps: identifying the research question, relevant studies, selecting studies, charting data, and collating, summarizing, and reporting results (11). The first and second authors (MD and DI) created the search formula, and the first author searched the database. Text screening and ultrasonography data charting for hemiplegic shoulders were performed by 3 authors (MD, DI, and MK).

### Article selection

This scoping review included original articles that used ultrasonography to evaluate shoulder soft-tissue injuries in patients with stroke. The first author reviewed all titles, abstracts, and full-text papers. After screening the titles and abstracts, the following articles were excluded: (*i*) those that used ultrasonography as a treatment; (*ii*) those that used ultrasonography to evaluate an intervention (e.g., injection, taping training, physiotherapy, neuromuscular electrical stimulation, peripheral nerve stimulation, or electroacupuncture); (*iii*) those that did not use ultrasonography; (*iv*) those that used ultrasonography for areas other than the shoulder; (*v*) those that did not include stroke; (*vi*) editorials, commentaries, conference abstracts, letters, reviews, and case reports; and (*vii*) those not written in English or Japanese. After reviewing the full texts of the articles, we excluded those that did not assess soft-tissue injuries or did not use ultrasonography to diagnose hemiplegic shoulder pain.

A primary literature search was conducted using the PubMed and ICHUSI databases for studies published between 1966 and May 2023. The literature search conducted in May 2023 in MEDLINE used the following MeSH terms: (“stroke”[MeSH Terms] OR “stroke”[All Fields]) AND (“shoulder”[All Fields] OR “soft-tissue injury”[All Fields] OR “soft-tissue injuries”[All Fields]) AND (“diagnostic imaging”[MeSH Terms] OR “diagnostic imaging”[All Fields] OR “ultrasonography”[MeSH Terms] OR “ultrasonography”[All Fields] OR “ultrasonics”[MeSH Terms] OR “ultrasonics”[All Fields] OR “ultrasound”[All Fields] OR “ultrasounds”[All Fields]).

### Data extraction and summarizing

The first author extracted and recorded relevant information that matched the study aim, including study design, study phase, evaluation period, sample size, classification, ultrasonographic methods, and location of soft-tissue injuries. A study phase of less than 1 month was defined as acute, 1 to 3 months as subacute, and more than 3 months as chronic. Ultrasonographic methods include using the ultrasonographic transducer, evaluation of patient position, frequency, and assessment sites. Soft-tissue injury locations include tear/effusion/tendinitis of the long head of the biceps (LHB) tendon; tear/tendinitis of the subscapularis (SSC), supraspinatus (SSP), infraspinatus (ISP), and teres minor tendon (TM); and bursitis of the subacromial-subdeltoid (SA-SD). The data were compiled into a single spreadsheet and imported into Microsoft Excel 2021 (Microsoft^®^ Excel for MAC; Microsoft Corp, Redmond, WA, USA).

## RESULTS

### Selected studies and overview

**Fig. 1:**
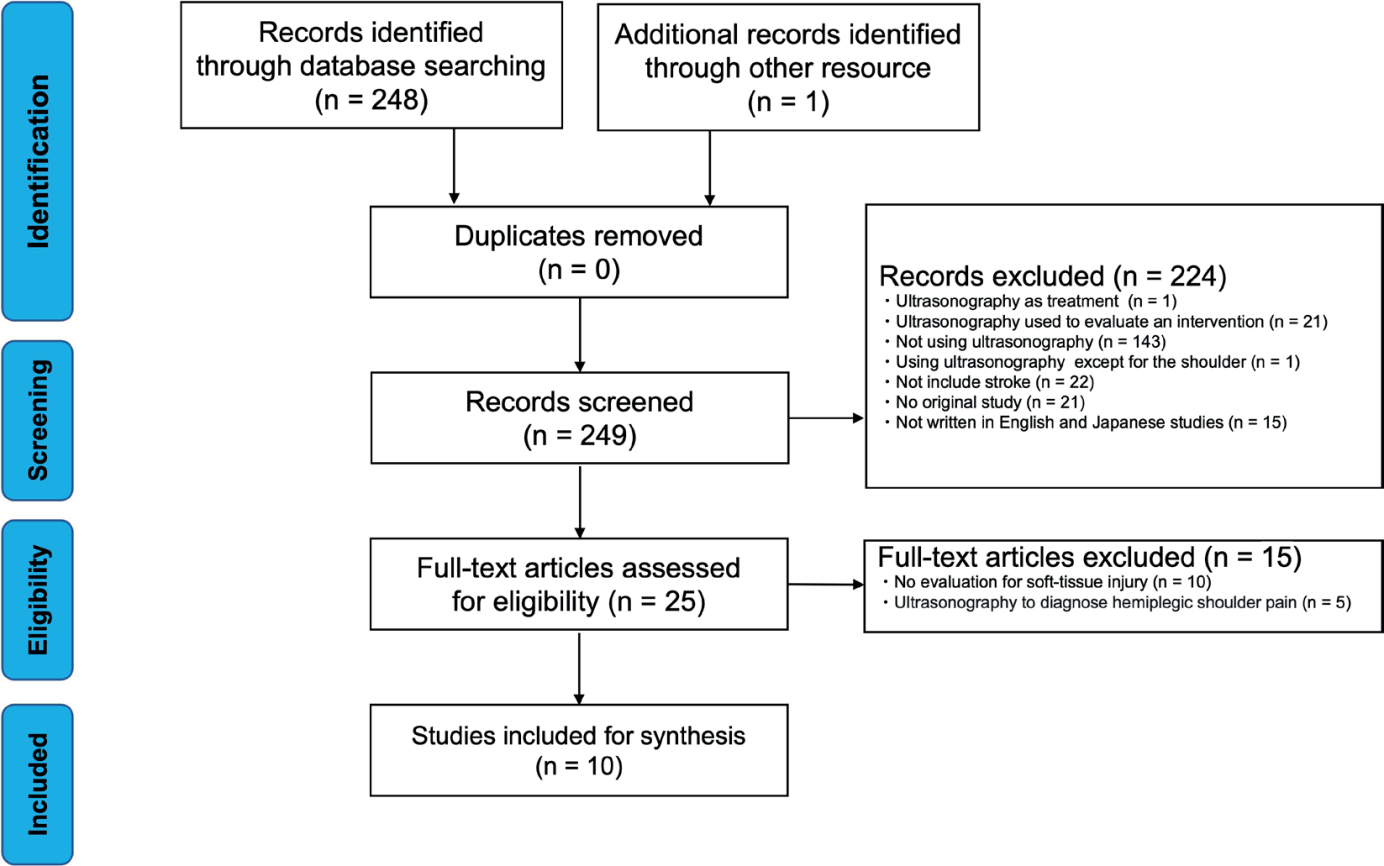
Preferred Reporting Items for Systematic Reviews and Meta-Analysis (PRISMA) extension for Scoping Reviews flow diagram.

The flow from identification to final inclusion is shown in Fig. 1. A total of 249 titles were retrieved, and 25 original studies that met the eligibility criteria were selected after duplicates were removed and screened. The reasons for exclusion were as follows: studies that used ultrasonography as treatment (*n* = 1); studies that used ultrasonography to evaluate an intervention (*n* = 21); studies that did not use ultrasonography (*n* = 143); studies that used ultrasonography for areas other than the shoulder (*n* = 1); studies that did not include stroke (*n* = 22); editorials, commentaries, conference abstracts, letters, reviews, and case reports (*n* = 21); and studies not written in English or Japanese (*n* = 15). Secondary screening excluded studies that did not assess soft-tissue injuries (*n* = 10) or did not use ultrasonography to diagnose hemiplegic shoulder pain (*n* = 5), and 10 publications were finally included.

An overview of these studies is presented in Table I. Among the 10 studies included, 4 were categorized as cohort studies (16, 18–20), 5 were cross-sectional studies (8, 12, 14, 15, 17), and 1 was a case-control study (13). Among the 7 cohort studies, 2 examined time-course changes in soft-tissue injuries at multiple time points (18, 19). Among all studies, 3 were conducted in the acute phase (8, 15, 19), 2 in the acute to subacute phase (17, 20), 2 in the acute to chronic phase (13, 16), 1 in the subacute to chronic phase (18), and 2 were uncertain (12, 14). The total and median sample sizes were 558 and 56 participants, respectively (interquartile range [IQR]: 27–100).

**Table I:** Characteristics of the included studies

Reference	Study design	Study phase and evaluation period	Sample size	Classification	Ultrasonographic methods	Soft-tissue injury locations
Xu et al., 2022 (12)	Cross-sectional study	Uncertain	100	(i) Pain group (ii) Non-pain group	Frequency: 6–15 MHz. Probe: uncertain Position: uncertain Assessment sites: LHB tendon SSP tendon SSC tendon SA-SD bursa	LHB tendon tear/effusion/inflammation SSP tendon tear/effusion/inflammation SSC tendon tear/effusion/inflammation SA-SD bursa effusion/inflammation
Idowu et al., 2017 (13)	Case-control study	Acute to chronic	45	(i) Hemiplegic side (ii) Non-hemiplegic side	Frequency: 7–12 MHz Probe: linear Position: sitting position Assessment sites: LHB tendon SSC tendon SSP tendon ISP tendon SA-SD bursa	LHB tendon effusion/tendinitis/degeneration SSC tendon tendinitis SSP tendon tear/tendinitis SA-SD bursitis
Idowu et al., 2017 (14)	Cross-sectional study	Uncertain	45	(i) Hemiplegic side (ii) Non-hemiplegic side	Frequency: 7–12 MHz Probe: linear Position: sitting position Assessment sites: LHB tendon SSC tendon SSP tendon ISP tendon SA-SD bursa	LHB tendon lesion SSC tendon lesion SSP tendon lesion ISP tendon lesion SA-SD bursa effusion
Hanayama et al., 2016 (15)	Cross-sectional study	Acute	27	(i) Pain group (ii) Non-pain group	Frequency: uncertain Probe: uncertain Position: sitting position Assessment sites: LHB tendon SSC tendon SSP tendon ISP tendon	LHB tendon lesion SSC tendon lesion SSP tendon lesion ISP tendon lesion
Kim et al., 2014 (16)	Cohort study	Acute to chronic	94	None	Frequency: 5–13 MHz Probe: linear Position: uncertain Assessment sites: LHB tendon SSC tendon SSP tendon ISP tendon SA-SD bursa	LHB tendon effusion SSC tendon tear/tendinitis SSP tendon tear/tendinitis ISP tendon tear/tendinitis SA-SD bursa effusion
Huang et al., 2012 (17)	Cross-sectional study	Acute to subacute	39	None	Frequency: 5–12 MHz Probe: linear Position: sitting position Assessment sites: LHB tendon SSP tendon ISP tendon SSC tendon	LHB tendon tear/tendinitis SSP tendon tear/tendinitis ISP tendon tear/tendinitis
Pong et al., 2012 (18)	Cohort study	Subacute to chronic	76	None	Frequency: 5–12 MHz Probe: linear Position: uncertain Assessment sites: LHB tendon SSC tendon SSP tendon ISP tendon SA-SD bursa	LHB tendon tear/effusion/tenosynovitis/tendinitis SSC tendon tear/tendinitis SSP tendon tear/tendinitis SA-SD bursitis/effusion
Huang et al., 2010 (8)	Cross-sectional study	Acute	57	(i) Severe paresis group (BRS Stage I to III) (ii) Mild paresis group (BRS Stage IV to VI)	Frequency: 5–12 MHz Probe: linear Position: uncertain Assessment sites: LHB tendon SSC tendon SSP tendon ISP tendon SA-SD bursa	LHB tendon tear/effusion/tenosynovitis/tendinitis SSC tendon tear/tendinitis SSP tendon tear/tendinitis SA-SD bursitis
Pong et al., 2009 (19)	Cohort study	Acute (i) Admission (ii) 2 weeks after rehabilitation	34	(i) Severe paresis group (BRS stage I to III) (ii) Mild paresis group (BRS Stage IV to VI)	Frequency: 5–10 MHz Probe: linear Position: uncertain Assessment sites: LHB tendon SSC tendon SSP tendon ISP tendon SA-SD bursa	LHB tendon effusion/tendinitis SSC tendon tendinitis SSP tendon tendinitis SA-SD bursa effusion
Park et al., 2007 (20)	Cohort study	Acute to subacute	41	None	Frequency: 5–12 MHz Probe: linear Position: sitting position Assessment sites: LHB tendon SSP tendon SSC tendon ISP tendon TM tendon SA-SD bursa	LHB tendon effusion SSC tendon tear/tendinitis SSP tendon tear/tendinitis ISP tendon tear/tendinitis SA-SD bursal effusion

ISP: infraspinatus; LHB: long head of the biceps; SA-SD: subacromial-subdeltoid; SSC: subscapularis; SSP: supraspinatus; TM: teres minor.

### Ultrasonographic methods

Among all studies, 9 clarified the frequency of ultrasonography (8, 12–14, 16–20), and 1 did not (15). In 6 studies, the ultrasonographic frequencies included the 5–7 MHz range (8, 16–20). A linear transducer was used in 8 studies (8, 13, 14, 16–20) and was uncertain in 2 (12, 15). The evaluation position was sitting in 5 studies (13–15, 17, 20), and uncertain in 5 (8, 12, 16, 18, 19). The assessment sites included the SSP tendon in 10 studies (8, 12–20), followed by the LHB tendon in 10 (8, 12–20), the SSC tendon in 10 (8, 12–20), the ISP tendon in 9 (8, 13–20), the SA-SD bursa in 8 (8, 12–14, 16, 18–20), and the TM tendon in 1 (20).

### Location of soft-tissue injuries

The locations of soft-tissue injury findings included SSP tendon tear/tendinitis in 10 studies (8, 12–20), LHB tendon effusion/tendinitis in 10 (8, 12–20), SA-SD bursitis in 8 (8, 12–14, 16, 18–20), SSC tendon tear/tendinitis in 10 (8, 12–20), and ISP tendon tear/tendinitis in 6 (8, 14–17, 20).

## DISCUSSION

This scoping review aimed to identify existing ultrasonographic methods and the locations of soft-tissue injuries in studies using ultrasonography to assess shoulder soft-tissue injuries in patients with stroke.

Few previous studies have used ultrasonography to assess shoulder soft-tissue injuries in patients with stroke. Although we used only 3 search formulae (stroke, shoulder, and ultrasonography), only 10 studies were included. Most excluded studies used ultrasonography to evaluate interventions, such as injections and neuromuscular electrical stimulation, and did not examine shoulder soft-tissue injuries. Therefore, our results indicate that ultrasonography is not commonly used to assess shoulder soft-tissue injuries in patients with stroke, possibly owing to the skills required for ultrasonographic examination. The results of any imaging technique depend on the examiner’s skill (21); acquiring imaging skills requires a long learning period (22). Furthermore, the reliability and validity of ultrasonographic shoulder soft-tissue injury assessment remain to be determined. However, ultrasonography is useful because it provides immediate and quantitative assessments. Therefore, further research is needed to promote the use of ultrasonography in evaluating shoulder soft-tissue injuries in patients with stroke.

This review showed that the frequency, probe of the ultrasonographic transducer, and evaluation position in shoulder soft-tissue injury assessment in patients with stroke were generally consistent. More than half of the studies used linear transducers and sitting positions, with frequencies of 5–7 MHz. A systematic review summarizing 38 cohort studies of orthopaedic shoulder soft-tissue disorders reported that the frequency was 5–7 MHz (25 studies), and the probe was a linear transducer (31 studies) (7). Furthermore, the sitting position was appropriate for patients with orthopaedic shoulder disease (22). These findings are consistent with those of the present study in patients with stroke. A 5–7.5 MHz frequency is considered most suitable for assessing deep shoulder structures (23). A linear transducer is most suitable for extensive shoulder examination (24). The sitting position was the most appropriate for evaluation because the arm could be externally rotated and placed behind the patient’s back to evaluate LHB, SSC, SSP, and ISP tendons (7). These trends were independent of the disease. Therefore, an ultrasonographic method similar to that used for orthopaedic shoulder soft-tissue disorders should be used to assess shoulder soft-tissue injuries in patients with stroke.

However, assessment sites differed slightly between stroke and orthopaedic disorders. A systematic review of orthopaedic shoulder soft-tissue disorders reported that standardized examination protocols include the assessment of the LHB, SSC, SSP, and ISP tendons (7). In our findings for patients with stroke, the common assessment sites were the SSP tendon, the LHB tendon, the SSC tendon, the ISP tendon, and the SA-SD bursa. The LHB, SSC, SSP, and ISP tendons were consistent with orthopaedic shoulder soft-tissue disorder assessments, whereas the SA-SD bursa was examined more frequently in patients with stroke. This may be because SA-SD bursitis is associated with hemiplegic shoulder pain. A previous study examining soft-tissue injuries with ultrasonography in patients with hemiplegic shoulder pain reported that SA-SD bursa effusion was the most common abnormal finding (25). Furthermore, SA-SD bursa effusion is a risk factor for partial rotator cuff tears (26). Therefore, the SA-SD bursa is an essential assessment site in patients with stroke.

Additionally, among the rotator-cuff muscles essential to the hemiplegic shoulder, the TM has seldom been assessed. This is possibly because TM tendon injuries are extremely rare in patients with orthopaedic shoulder soft-tissue disorders. A previous study in patients with orthopaedic shoulder soft-tissue disorders reported that 90.8% of TM tendons were normal (27). Accordingly, standardized examination protocols for orthopaedic shoulder soft-tissue disorders did not include the TM tendon. However, rotator cuff injuries are commonly observed in patients with stroke. In a previous study using ultrasonography on patients with hemiplegic shoulder pain, rotator cuff injuries were observed in 59.4% (20). The TM assists in lateral rotation, horizontal abduction, and arm abduction, helping to stabilize the glenohumeral joint (24). Post-stroke hemiplegia impairs the dynamic control and support function of the rotator cuff, and TM function may also be affected. Therefore, evaluating TM function in patients with stroke is important, and ultrasonography may be a valid method. Further research is needed to study the prevalence of TM injuries in patients with stroke.

The most common locations of shoulder soft-tissue injuries were the LHB tendon effusion/tendinitis and SSP tendon tear/tendinitis. The high frequency of LHB tendon effusion/tendinitis can be attributed to spasticity. The elbow flexors (biceps brachii) are the most spastic muscles in patients with stroke (4). Spastic muscles have a reduced optimal fibre length owing to muscle shortening after stroke (28), and shortened muscle tendons are prone to injury from extension, leading to repair at the anatomical footprint (29). Accordingly, the most common location of soft-tissue injury might be LHB tendon effusion/tendinitis.

The high frequency of SSP tendon effusion/tendinitis may be attributed to flaccid paralysis. Flaccid paresis is observed in most limbs during the early stages of stroke (30), leading to shoulder subluxation. Shoulder subluxation is common, with a previous study reporting a 56–81% occurrence in patients with stroke (30). Additionally, shoulder subluxation causes soft-tissue damage (31). Flaccid paralysis may cause humeral head migration in the shoulder joint, leading to soft-tissue injuries due to overstretching of the capsule, tendons, and ligaments (5). Therefore, shoulder subluxation owing to flaccid paralysis may have contributed to soft-tissue injuries.

Furthermore, SSP tendon effusion or tendinitis may be associated with shoulder impingement syndrome. Shoulder impingement syndrome is a pathology with distinct clinical symptoms, such as pain during attempted use of the shoulder (7). Normal shoulder joint motion can prevent impingement of the supraspinatus tendon between the humeral head and the acromion by upward rotation of the scapula, which elevates the coracoacromial arch and allows the glenoid to maintain firm opposition to the humeral head (32). Conversely, the hemiplegic shoulder often cannot maintain the scapular position because of muscle paresis, such as the trapezius, levator scapulae, rhomboid minor, rhomboid major, and serratus anterior (33); therefore, the humeral head may ride up on the glenoid surface during shoulder flexion or abduction (38). Consequently, the hemiplegic shoulder may cause shoulder impingement syndrome of the SSP tendon between the humeral head and the acromion (32). Shoulder impingement syndrome is highly prevalent and occurs in 57.8% of stroke survivors with shoulder pain (34). Accordingly, a common form of soft-tissue injury might be SSP tendon effusion/tendinitis.

That is, these may be secondary effects of stroke symptoms, such as spasticity and paralysis. As spasticity and paralysis are common symptoms in patients with stroke, they are well known to cause pain (35) and functional impairment (36). Therefore, clinical assessments of spasticity and paralysis are abundant, and their time-course changes have been well documented. However, a general clinical evaluation of the secondary effects of spasticity and paralysis on bones and muscles does not exist. However, ultrasonography can assess the bones and muscles of hemiplegic shoulders after a stroke. Therefore, ultrasonography can be a useful tool for evaluating the secondary effects of post-stroke neurological symptoms on anatomical structures.

This scoping review had some limitations. First, only original human studies published in English and Japanese were included; commentaries, conference abstracts, case reports, and reviews were excluded. However, because we confirmed the PubMed ID for each study, the risk of data duplication was minimized. Furthermore, our search strategy focused only on 2 databases and did not include grey literature; therefore, the risk of publication bias was significant. Additionally, we could not map the study design and phase because only a few studies were selected. However, this scoping review was conducted in accordance with the PRISMA-ScR guidelines.

In conclusion, this study is the first scoping review to map studies using ultrasonography to assess shoulder soft-tissue injuries in patients with stroke and to identify ultrasonographic methods and the injury locations. Further studies using ultrasonography to evaluate shoulder soft-tissue injuries in patients with stroke may help standardize assessment methods and improve clinical practice.
